# Monitoring the Right Collection: The Central Cholinergic Neurons as an Instructive Example

**DOI:** 10.3389/fncir.2017.00031

**Published:** 2017-04-27

**Authors:** Katalin Sviatkó, Balázs Hangya

**Affiliations:** ^1^Lendület Laboratory of Systems Neuroscience, Department of Cellular and Network Neurobiology, Institute of Experimental Medicine – Hungarian Academy of SciencesBudapest, Hungary; ^2^János Szentágothai Doctoral School of Neurosciences, Semmelweis UniversityBudapest, Hungary

**Keywords:** cell types, optogenetics, connectivity, cholinergic, Alzheimer’s disease

## Abstract

Some neurons are more equal than others: neuroscience relies heavily on the notion that there is a division of labor among different subtypes of brain cells. Therefore, it is important to recognize groups of neurons that participate in the same computation or share similar tasks. However, what the best ways are to identify such collections is not yet clear. Here, we argue that monitoring the activity of genetically defined cell types will lead to new insights about neural mechanisms and improve our understanding of disease vulnerability. Through highlighting how central cholinergic neurons encode reward and punishment that can be captured by a unified framework of reinforcement surprise, we hope to provide an instructive example of how studying a genetically defined cell type may further our understanding of neural function.

## Introduction

In 2014 Mahlon DeLong and Alim-Louis Benabid got the Lasker prize for deep brain stimulation, serving as a vivid reminder that one of neuroscience’s greatest clinical success stories was based on diligent – and often tedious – recording of hundreds of basal ganglia neurons from monkeys (DeLong, 1969, 1971; Wichmann and Delong, 2002, 2006). Although those recordings were done in the seventies and eighties, similar electrophysiology experiments are still vital today. For instance, amidst the gloomy mood ensuing the disappointing outcome of the phase 3 clinical trials of anti-amyloid antibodies for treating Alzheimer’s disease, a commentary in Nature asserted that “the biggest clues (for therapy) will come from monitoring collections of neurons" (Kosik, 2013).

As often as not, the devil is in the details: what defines a ‘collection’ of neurons? The way we choose which neuronal pool to study will determine the interpretation and impact of our experiments – and it is usually far from trivial to pick the ‘right’ collection. Indeed, sampling random sets of brain cells results in what was coined the neuronal ‘response zoo’: the perplexing complexity of individual activities lacking clear boundaries and not yielding clear answers.

## Multiple ways of Defining ‘Collections’ of Neurons

**FIGURE 1:**
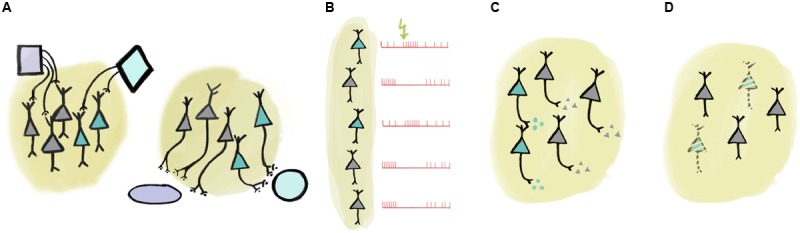
**Multiple ways of defining ‘collections’ of neurons. (A)** Collections of neurons may be defined by input or output connectivity. **(B)** Response to external or internal events may identify a set of functionally similar neurons. **(C)** Neurochemical identity (e.g., neurotransmitter profile) defines cell types. **(D)** Certain cell types may be specifically linked to disease.

There are multiple ways of defining more specific – and thus often more useful – collections (**Figure 1**). These collections may encode ‘low-level’ behavioral information in the form of computationally tractable behavioral variables, like reward expectation, subjective value, expected and unexpected uncertainty, temporal anticipation, etc. (Schultz et al., 1997; Yu and Dayan, 2005;

Dayan, 2012). For instance, we can identify groups of neurons that may mediate specific brain functions by selecting them according to their connectivity (**Figure 1A**). One nice example of such dissociation between hodology-based collections was demonstrated between midbrain dopaminergic neurons with different projection targets (Parker et al., 2016). It was found that while both collections coded reward prediction errors as typical for dopaminergic neurons (Schultz et al., 1997), those projecting to the dorsomedial striatum responded more to contralateral choice, whereas dopamine neurons with projections to the nucleus accumbens responded prominently to reward consumption and reward-predicting cues (Parker et al., 2016). Another study defined collections of midbrain dopaminergic neurons based on their afferent rather than their efferent connectivity (Dautan et al., 2016). Dautan et al. (2016) demonstrated that dopaminergic neurons receiving cholinergic input from the laterodorsal tegmentum were excited, whereas those innervated by the pedunculopontine cholinergic neurons were inhibited by aversive stimuli. The prefrontal cortex (PFC) also represents an important hub of the reward processing circuitry. Projection-specific coding has recently been demonstrated in the PFC: corticostriatal neurons show excitatory, whereas corticothalamic projection neurons develop inhibitory responses to reward-predicting cues during learning (Otis et al., 2017). Bidirectional projection neurons between the PFC and the amygdala also carry out target-specific functions both from PFC to amygdala (Courtin et al., 2013) and vice versa (Senn et al., 2014). In addition, functional differences between corticopontine and commissural PFC neurons were also demonstrated (Dembrow et al., 2010).

Responses to sensory stimulation as well as other characteristic response properties may also be employed to define functional collections (**Figure 1B**). For instance, there is an association between tone-responsiveness and disinhibition by VIP interneurons in the auditory cortex principal neuron population (Pi et al., 2013). As another example, putative non-cholinergic neurons of the basal forebrain that show stereotypical burst responses to reward-predictive cues share a number of functional properties (Lin and Nicolelis, 2008; Avila and Lin, 2014). Naturally, these defining features may be strongly correlated, in that cells with shared inputs likely have similar response properties and may have overlapping efferent connectivity (Varga et al., 2010; Gielow and Zaborszky, 2017).

One particularly useful trait for cell type identification is by neurochemical profile (**Figure 1C**). For instance, neurons of a given area that use different neurotransmitters are often distinguished by local and long-range connectivity as well as activity patterns in response to external stimuli or internal variables, lending credibility to the notion of treating them as a functional ‘collection’ (Varga et al., 2010; Do et al., 2016; Gielow and Zaborszky, 2017). Nevertheless, neurotransmitters alone may fall short in delineating practical functional groups. In case of glutamatergic neurons, combination with efferent connectivity (Dembrow et al., 2010; Otis et al., 2017) or more detailed neurochemical identification (Ye et al., 2016) may prove sufficient. In GABAergic interneurons, expression of other genetically defined markers like calcium-binding proteins or specific receptors provide effective genetic handles on functional collections. For instance, interneurons that express the calcium-binding protein parvalbumin show functional homogeneity in PFC (Hartwich et al., 2009; Massi et al., 2012; Kvitsiani et al., 2013; but see Lagler et al., 2016), motor cortex (Isomura et al., 2009), somatosensory cortex (Sachidhanandam et al., 2016), visual cortex (Atallah et al., 2012; Lee et al., 2012; Wilson et al., 2012), auditory cortex (Moore and Wehr, 2013), or hippocampus (Lapray et al., 2012; Viney et al., 2013). An association between projection targets, genetic labels (NPAS4-expression) and coding properties during behavior was elegantly demonstrated in the PFC recently (Ye et al., 2016).

The notion of genetically defined functional collections is underscored by observations of specific loss of neurotransmitter-defined cell types in many neurological diseases (**Figure 1D**). For instance, Alzheimer’s disease is characterized by the gradual loss of basal forebrain cholinergic neurons (Whitehouse et al., 1982; Arendt and Bigl, 1986). While the atrophy of the same cells is associated with the cognitive symptoms developed in most Parkinson’s patients, the motor symptoms that define the disease are a result of the loss of dopaminergic innervation (Gratwicke et al., 2015). Other examples include the association between the dysfunction of parvalbumin-expressing cortical interneurons and schizophrenia (Sohal et al., 2009; McNally and McCarley, 2016), or narcolepsy caused by the loss of orexin-signaling (Lin et al., 1999; Peyron et al., 2000; Thannickal et al., 2000; Kohlmeier et al., 2013).

## Central Cholinergic Neurons as an Example Collection to Study

As mentioned above, Alzheimer’s disease is characterized by the gradual loss of basal forebrain cholinergic neurons. These neurons are situated at the bottom of the forebrain and send extensive projections to all cortical areas (Saper, 1984; Zaborszky et al., 2012, 2013; Do et al., 2016), releasing the neurotransmitter acetylcholine from their terminals. Experiments with selective ablation of cholinergic neurons or pharmacological blockade of acetylcholine suggest that these neurons mediate important cognitive functions, including learning and attention (Everitt and Robbins, 1997; Wrenn and Wiley, 1998; Parikh et al., 2007). Indeed, cognitive deficits in Alzheimer’s patients are strongly correlated with the extent of cholinergic cell loss (Whitehouse et al., 1982; Arendt and Bigl, 1986).

**FIGURE 2:**
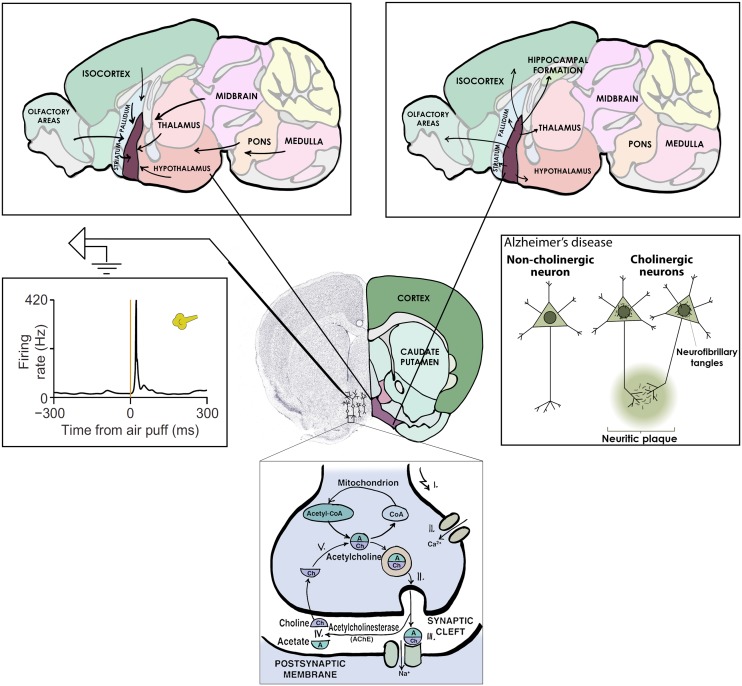
**Central cholinergic neurons as an example collection to study.** Basal forebrain cholinergic neurons release acetylcholine as neurotransmitter (bottom), share afferent (top left) and efferent (top right) connectivity, display uniform activation kinetics in response to aversive air puffs (middle left) and show cell type specific degeneration in Alzheimer’s disease (middle right).

Cholinergic neurons would thus appear like the ideal ‘collection’ of neurons to study; forming an anatomically and neurochemically distinct group, having important functions and a strong relevance to human disease (**Figure 2**). Given this, it may come as a surprise that the activity of forebrain cholinergic neurons during any behavior is largely unknown. The reason for this has been the lack of tools to specifically probe cholinergic neurons, which are intermingled with more numerous cell types, in awake behaving animals. The game changer is the recent advent of optogenetic methods that allow the expression of light-sensitive ion channels in genetically defined cell types – like those that express synthesizing enzymes for acetylcholine and are therefore cholinergic (Boyden et al., 2005; Rossi et al., 2011). Using optogenetics, one can activate cholinergic neurons by light and in this way identify them while the animal is awake and able to perform learned behaviors.

## Unique Activity Patterns of Cholinergic Neurons Hint at Functions in Cognition

We and others set out to tackle the long-unanswered question of when cholinergic neurons fire, to reveal how their activity patterns may support various aspects of normal and diseased cognition (Hangya et al., 2015; Harrison et al., 2016). To simultaneously probe aspects of learning and attention, the two leading theories of cholinergic function, we trained mice to respond to unpredictable quiet tones embedded in loud noise – a difficult sensory detection task that involves associative learning and demands sustained attention. To crack the identity of basal forebrain neurons, we turned to optogenetics: we rendered cholinergic neurons light-sensitive by combining the ChAT-Cre mouse line in which Cre-recombinase is expressed in cholinergic neurons with viral vectors that allow Cre-dependent expression of the light-sensitive channelrhodopsin. We placed microelectrodes into the basal forebrain, home to the cholinergic neurons, along with an optical fiber capable of delivering laser light into brain tissue. In our mice, cholinergic neurons, and only those, were activated by blue light, revealing their neurochemical identity. Finding the cholinergic cells still proved to be difficult, as over 90% of the neurons in the sparsely inhabited basal forebrain are non-cholinergic (Gritti et al., 2006).

Previous studies suggested that cholinergic neurons might be involved in controlling attention. First, lesions to basal forebrain cholinergic neurons caused impairments in tasks that require sustained attention like the five-choice serial reaction time task, during which rodents have to detect and respond to visual cues (Everitt and Robbins, 1997; Turchi and Sarter, 1997; Wrenn and Wiley, 1998; McGaughy et al., 2002; Dalley et al., 2004). Second, measures of acetylcholine release suggested that choline-transients appear in association with cue-detections in attention tasks (Parikh et al., 2007; Hasselmo and Sarter, 2011; Howe et al., 2013; Sarter et al., 2014). Third, cholinergic inputs to sensory cortices influence receptive field properties and stimulus tuning of sensory neurons, which may serve as a basis for attentional functions of the cholinergic system (Kilgard and Merzenich, 1998; Disney et al., 2007; Froemke et al., 2007; Herrero et al., 2008; Thiele et al., 2012). If cholinergic neurons controlled attention at a fast, trial-by-trial time scale, we would expect them to fire vigorously when mice anticipate the tone stimuli, that is, when they have to pay the most attention. Furthermore if these neurons indeed regulated attention levels, their stronger activity should foretell faster or more accurate behavioral responses. However, we found that cholinergic neurons, as a collection, did not show these patterns. Nevertheless, we observed gradual changes in the firing rate of cholinergic neurons throughout behavioral sessions, suggesting that attentional regulation by the cholinergic system demonstrated in previous studies may be mediated by slower modulation of cholinergic firing (Lee et al., 2005; Paolone et al., 2012; Teles-Grilo Ruivo et al., 2017).

On the other hand, basal forebrain cholinergic neurons were promptly activated by reward and punishment, with unexpected speed and precision (**Figure 2**). This finding is consistent with three studies in which calcium imaging or voltammetry was used to track cholinergic responses (Lovett-Barron et al., 2014; Harrison et al., 2016; Teles-Grilo Ruivo et al., 2017); however, the low temporal resolution of these methods prevented the appreciation of the speed of cholinergic firing. Our results are in line with an elegant recent report on the dissociation of tonic and phasic release of acetylcholine using choline-sensitive biosensors (Teles-Grilo Ruivo et al., 2017).

Moreover, we found that the extent of cholinergic activation was proportional to the unexpectedness, or ‘surprise,’ of the behavioral feedback: cells fired more to reward if it was delivered after an ambiguous auditory cue. Such activation patterns support the role of the cholinergic system in controlling learning: after behavioral feedback such as reward and punishment, there is a unique opportunity to form associations between the stimulus perceived, the action performed and the outcome received. This aspect of cholinergic neurons is notably similar to midbrain dopaminergic neurons, which represent reward prediction errors important for reinforcement learning (Schultz et al., 1997; Bayer and Glimcher, 2005). Nevertheless, reward prediction error is defined in the context of cued outcome tasks, therefore a difference in the behavioral paradigms studied prevents a direct comparison of the two cell types. We expect that understanding how these and other neuromodulatory cell types are coordinated to support learning will be an area of intense research in the near future (Ogawa et al., 2014; Varazzani et al., 2015; Matias et al., 2017).

## Conclusion

These are only the first steps toward understanding how functional collections of neurons may contribute to behavior. Nevertheless, cholinergic neurons can serve as an instructive example of a genetically defined cell type that broadcasts a computationally tractable behavioral variable. As a next step, it will be important to understand how such ‘low-level’ information coded by cell types is combined into behaviorally relevant complex information, probably mediated by ensembles composed of the appropriate cell types and interconnections. Eventually, the study of genetically defined cell types and cell type-ensembles may lead to one of those elusive clues for future clinical investigations of neurodegenerative diseases.

## Author Contributions

BH developed the idea, BH and KS conceived the manuscript, BH wrote the text and KS generated the figures.

## Conflict of Interest Statement

The authors declare that the research was conducted in the absence of any commercial or financial relationships that could be construed as a potential conflict of interest.
